# Improved enhancement in CT angiography with reduced contrast media iodine concentrations at constant iodine dose

**DOI:** 10.1038/s41598-018-35918-y

**Published:** 2018-11-30

**Authors:** Toon Van Cauteren, Gert Van Gompel, Koenraad H. Nieboer, Inneke Willekens, Paul Evans, Sven Macholl, Steven Droogmans, Johan de Mey, Nico Buls

**Affiliations:** 1 Departement of Radiology, Vrije Universiteit Brussel (VUB), Universitair Ziekenhuis Brussels (UZ Brussel), Laarbeeklaan 101, 1090 Brussels, Belgium; 2 Departement of Imaging R&D, GE Healthcare Life Sciences, Amersham, Buckinghamshire England; 3 Departement of Cardiology, Vrije Universiteit Brussel (VUB), Universitair Ziekenhuis Brussels (UZ Brussel), Laarbeeklaan 101, 1090 Brussels, Belgium

## Abstract

The study objective is to investigate the impact of a wide range of contrast media (CM) iodine concentrations on CT enhancement at constant total iodine dose (TID) and iodine delivery rate (IDR). Seven injection protocols, based on different iodine concentrations ranging from 120 to 370 mg I/mL, were assessed on 4 minipigs at a constant TID of 320 mg I/kg and IDR of 0.64 g I/s. Dynamic images were acquired on a clinical 64-slice MDCT scanner for 120 s with the abdominal aorta, vena cava inferior and liver parenchyma in the field-of-view. Maximal enhancement, time-to-peak and peak width were assessed. The enhancement curve characteristics were correlated with CM iodine concentration. In particular, CM with lower iodine concentrations yielded a significant increased maximal enhancement and peak width compared to the standard-of-care concentrations: e.g. in the aorta, 245 HU maximal enhancement and 9.2 s peak width with the 320 mg I/mL iodine concentration increased to 291 HU and 16.1 s with 160 mg I/mL. When maintaining a constant TID and IDR, by compensating injection rate and volume, injection of a CM with reduced iodine concentration results in a diagnostically beneficial higher maximal enhancement and longer enhancement peak duration.

## Introduction

Computed tomography (CT) has been an essential diagnostic tool in the clinic for decades. Continuous development of acquisition hardware, software and image reconstruction algorithms provided substantial image quality improvements e.g. in terms of spatial and temporal resolution^1^. The majority of clinical CT scans require the administration of iodine based contrast media (CM) for soft tissue differentiation. They are designed to increase the absorption of x-ray photons and enhance image contrast of blood vessels and well perfused tissues. Although these contrast media are frequently used in medical practice, they can cause contrast-induced nephropathy (CIN), which is associated with increased mortality in at risk patients with renal insufficiency^2–6^. Injection protocol parameters such as injection volume, injection rate, CM iodine concentration and saline chaser have an important impact on image quality. A standard clinical abdominal injection protocol administers 100 ml of contrast media, with an iodine concentration of 350 mg I/mL, at an injection rate of 4 ml/s for a 70 kg patient^7^. These injection protocol parameters have been the subject of many optimisation studies^8–18^. The majority of these studies focussed on investigating blood vessel or tissue enhancement as a function of two or three clinically used CM with different iodine concentrations^8–12^. Tissue enhancement is reported to depend on the injection protocol with the highest iodine dose because of the linear relation between iodine concentration in blood and the CT value^19^. However, despite lower iodine dose, two studies reported no difference in tissue enhancement between two CM with different iodine concentrations^11,12^.

An alternative approach to investigate the effect of CM with different iodine concentrations on CT enhancement, is to administer CM at a constant total iodine dose (TID (mg I)) and iodine delivery rate (IDR (mg I/s)). Several studies, using this approach on a selection of two or three CM, reported an improved CT enhancement in primary blood vessels^13–17^ and liver parenchyma^18^ when administering CM with the lower iodine concentrations. However, the underlying mechanisms that CM iodine concentration have on enhancement remains difficult to evaluate with clinical CT scan protocols in patient populations.

The objective of this preclinical animal study was to investigate the impact of the CM iodine concentration on CT enhancement at constant TID and IDR in a number of biologically relevant compartments. In contrast to previously published studies, this has been done in a within subject study design for a unique and extensive range of CM iodine concentrations varying from subclinical iodine concentrations of 120 mg I/mL to standard of care (SoC) concentrations of 320 and 370 mg I/mL. Also more detailed information on the enhancement patterns was obtained by using a modern generation CT scanner, allowing sub-second temporal resolution dynamic scans. The minipig model, equipped with a port-a-cath (PAC) unit to standardise the CM injections, allowed this high radiation dose dynamic CT scans which would be unethical in human patients. They also enable us to investigate the net effect of CM iodine concentration in a standardised and reproducible way without inter-patient variability.

## Results

**Figure 1:**
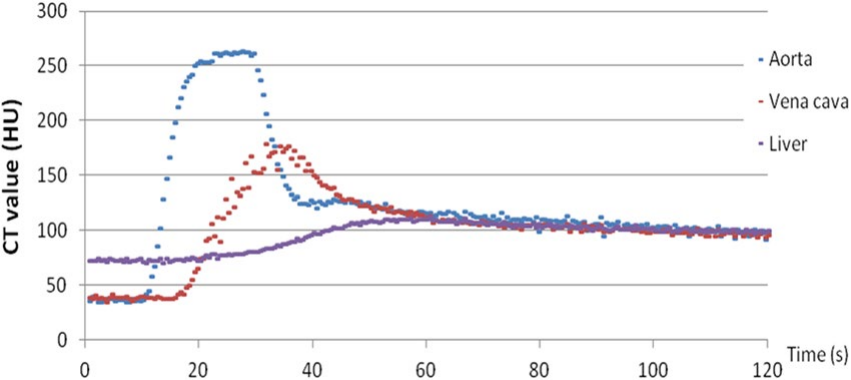
Example of time enhancement curves measured in the aorta (blue), vena cava (red) and liver parenchyma of an injection protocol based on CM with an iodine concentration of 160 mg I/mL.

**Figure 2:**
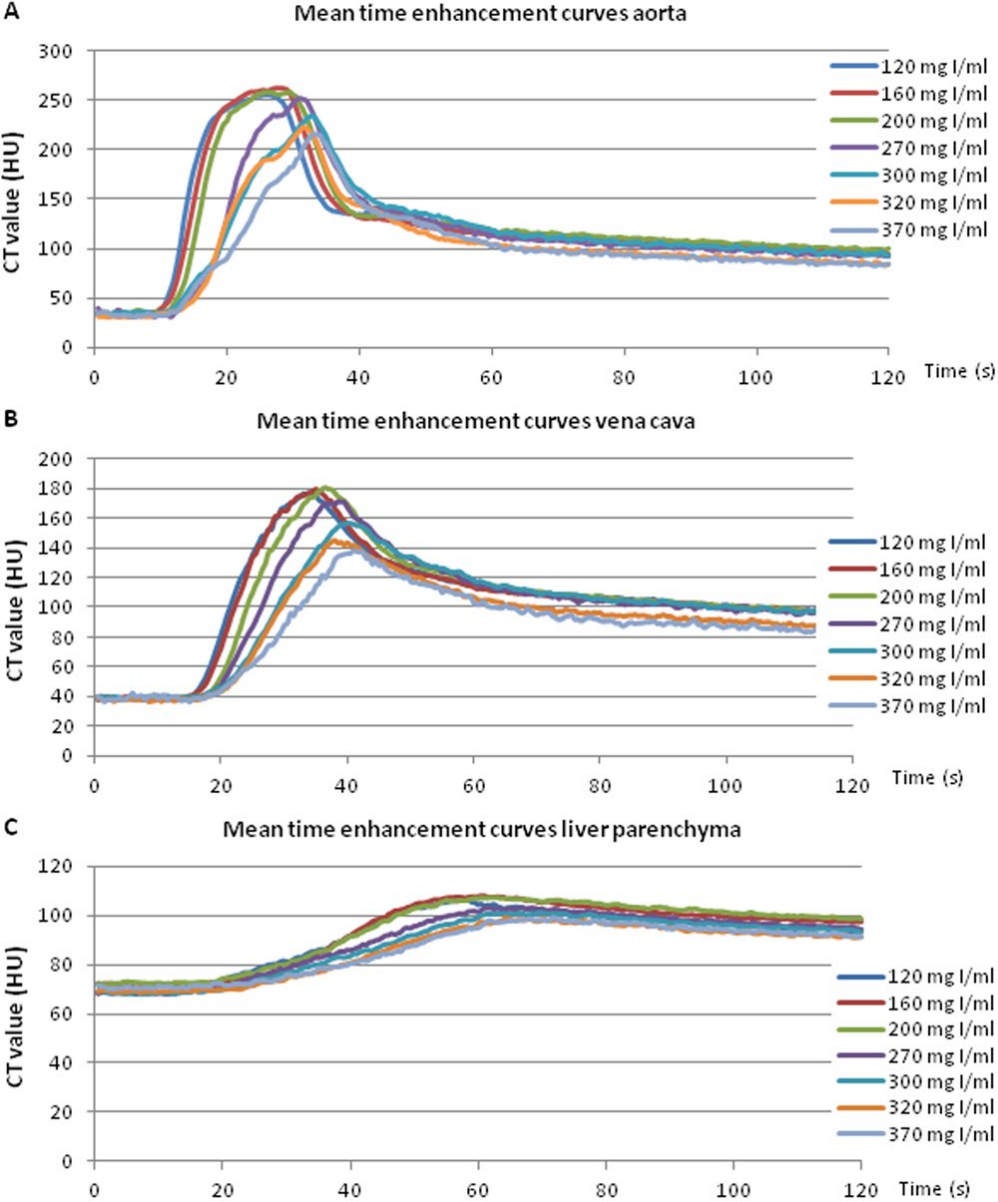
Mean time enhancement curves aorta (**A**), vena cava (**B**) and liver parenchyma (**C**) at constant TID (320 mg I/kg) and IDR (0.64 g I/s).

For each acquisition, time enhancement curves were obtained from the abdominal aorta, vena cava inferior and liver parenchyma. An example is given in Fig. 1 which presents the enhancement patterns for the 160 mg I/mL protocol in the considered ROIs. Figure 2 shows the mean time enhancement curves of each of the investigated injection protocols for the abdominal aorta (A), the vena cava inferior (B) and liver parenchyma (C), respectively.

### Aortic enhancement

**Figure 3:**
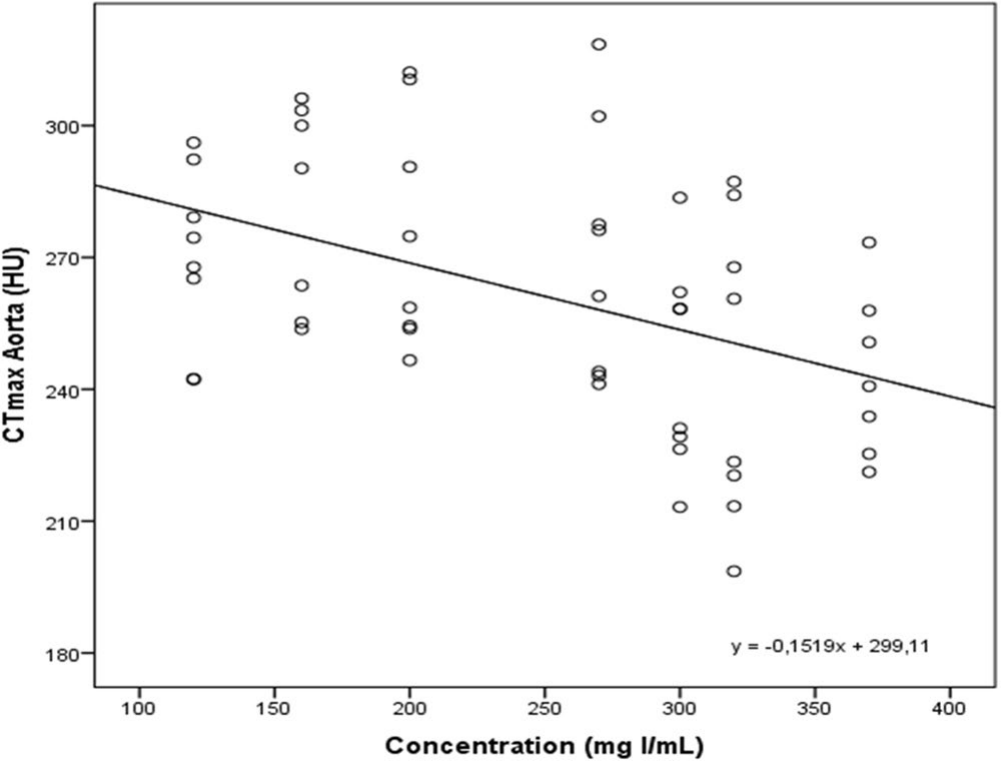
Negative correlation between maximal arterial enhancement and CM iodine concentration found with a Spearman Rank-Order Correlation test (r = 0.49, *p*-value < 0.0001).

**Figure 4:**
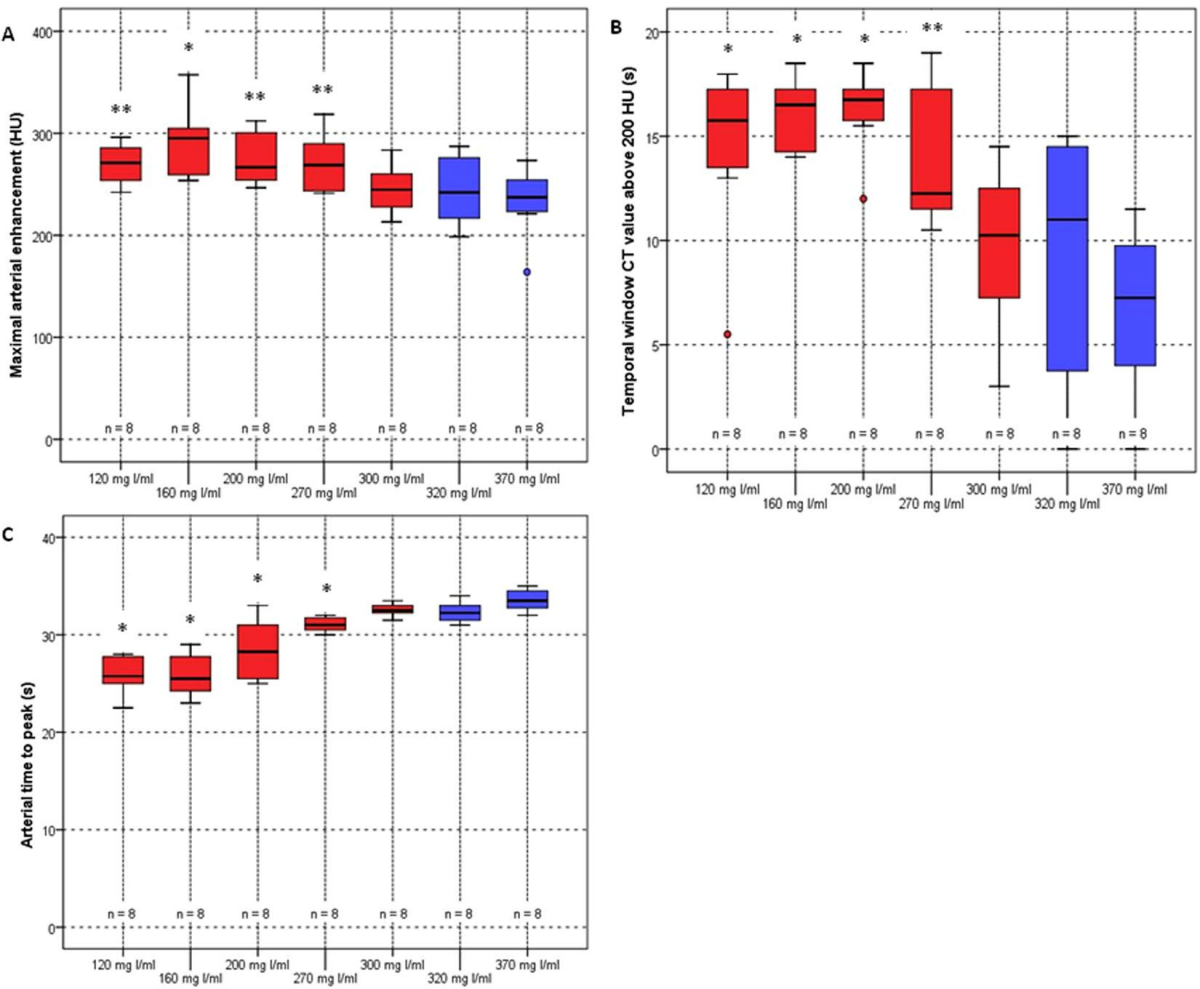
Boxplots of the maximal enhancement (**A**), the temporal window in which the CT signal remained above 200 HU (**B**) and the time to peak (**C**) in the abdominal aorta. The solid line in the box represents the median value and the upper and lower bars represent the first and third quartiles, respectively. The whiskers represent the 95% confidence interval, outliers are identified by a circle. The blue boxplots represent the SoC iodine concentrations, the red boxplots the diluted iodine concentrations. *Indicates significant difference compared to SoC CM with iodine concentrations of 320 and 370 mg I/mL (*p* < 0.05) while **indicates significant difference compared to SoC CM with iodine concentration of 370 mg I/mL (*p* < 0.05).

**Figure 5:**
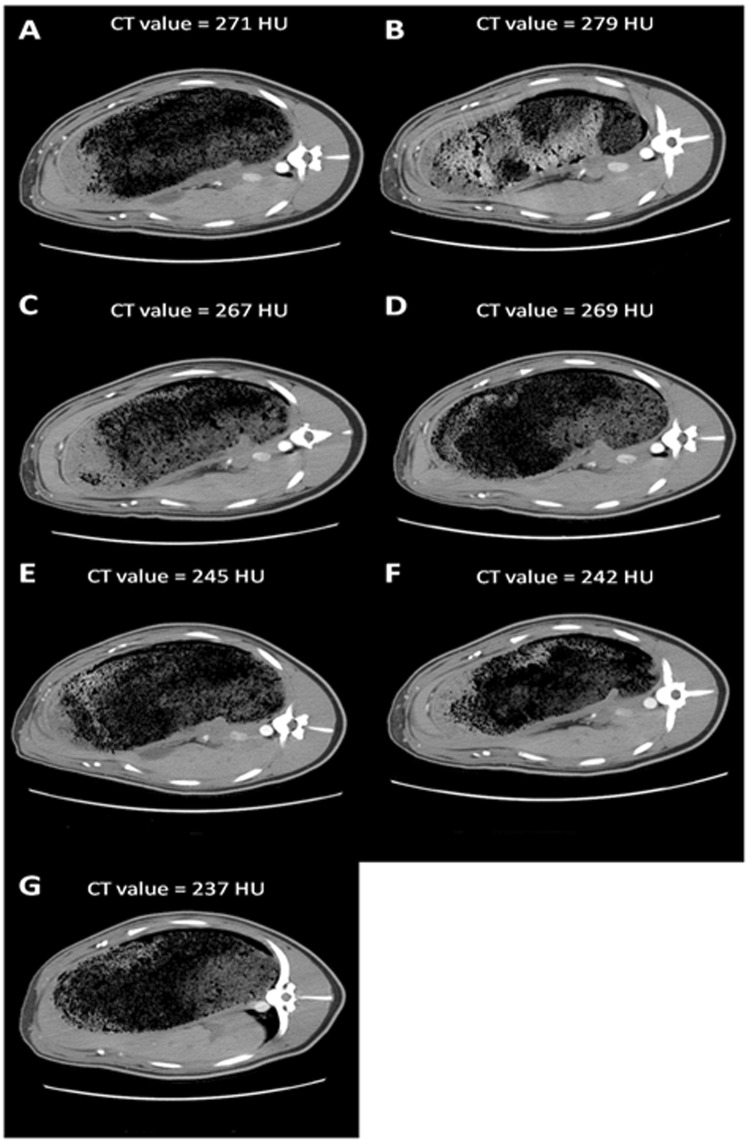
Example of axial images at the hepatic level of the different injection protocols at their maximal arterial enhancement, show decreasing enhancement of the abdominal aorta (white arrow) for increasing CM concentrations ((**A**) 120 mg I/mL, (**B**) 160 mg I/mL, (**C**) 200 mg I/mL, (**D**) 270 mg I/mL, (**E**) 300 mg I/mL, (**F**) 320 mg I/mL and (**G**) 370 mg I/mL). (Window = 400, Level = 40). Difference in enhancement can be visually appreciated.

At constant TID and IDR, a negative correlation between maximal arterial enhancement and CM iodine concentration was found (r = 0.46, *p*-value < 0.0001) (Fig. 3). Improved arterial enhancement of the diluted CM was observed compared to the CM with SoC iodine concentrations (320 and 370 mg I/mL). The maximal enhancement (CT_max_) of injection protocols with iodine concentrations of 120, 160, 200 and 270 mg I/mL was significantly higher compared to the CT_max_ of the SoC iodine concentration of 370 mg I/mL (*p*-values between 0.005 and 0.021) (Figs 2A and 4A and Table 1). Also, a significant difference was found between the CT_max_ of injection protocols with iodine concentrations of 160 and 320 mg I/mL (*p* = 0.027). A trend towards increased enhancement was observed with reduced iodine concentrations at equal TID and IDR. This is illustrated in Fig. 5, containing axial images of the different injection protocols at their maximal arterial enhancement of each CM iodine concentration. A gradual decrease of enhancement in the abdominal aorta could be observed with increasing iodine concentration based injection protocols.

**Table 1:** Mean values (N = 8) of each Figure of Merit for the aorta with corresponding 95% confidence intervals.

CM iodine concentration (mg I/mL)	CT_max_ (HU)	∆t_>200_ (s)	TTP (s)
120	270.0** (253.1–286.8)	14.6*^,^** (11.2–17.9)	25.9*^,^** (24.4–27.5)
160	291.2*^,^** (262.4–320.0)	16.1*^,^** (14.6–17.5)	25.9*^,^** (24.0–27.7)
200	275.2** (253.2–297.1)	16.3*^,^** (14.6–17.9)	28.4*^,^** (25.8–31.1)
270	270.5** (246.4–294.5)	13.9** (11.1–16.8)	31.1*^,^** (30.4–31.7)
300	245.3 (225.5–265.1)	9.7 (6.5–12.8)	32.6 (32.0–33.1)
320	244.5 (215.7–273.2)	9.2 (4.3–14.1)	32.3 (31.4–33.2)
370	233.4 (205.8–260.9)	6.7 (3.4–10.0)	33.6 (32.7–34.4)

*Indicates significant difference compared to SoC CM with iodine concentration of 320 mg I/mL (*p* < 0.05).

**Indicates significant difference compared to SoC CM with iodine concentration of 370 mg I/mL (*p* < 0.05).

The arterial peak width (∆t_>200_) for which CT signal remained above 75 percent from the CT_max_ or 200 HU, was significantly larger for 120, 160 and 200 mg I/mL compared to the SoC iodine concentrations of 320 and 370 mg I/mL (*p*-values between 0.001 and 0.027) (Fig. 4B and Table 1). For the iodine concentration of 270 mg I/mL, ∆t_>200_ was also significant larger compared to the SoC iodine concentration of 370 mg I/mL (*p* = 0.003).

The time to peak (TTP), was significantly lower for the iodine concentrations of 120, 160, 200 and 270 mg I/mL compared to the SoC concentrations of 320 and 370 mg I/mL (*p*-values between 0.001 and 0.019) (Fig. 4C and Table 1).

### Inferior vena cava enhancement

Again, a negative correlation between maximal enhancement in the vena cava inferior and CM iodine concentration was found (r = 0.57, *p*-value < 0.0001) at constant TID and IDR. The CT_max_ values of the iodine concentrations of 120, 160, 200 and 270 mg I/mL were significantly higher compared to the CT_max_ of the SoC CM with 370 mg I/mL (*p*-values between 0.002 and 0.005) (Fig. 2B and Table 2). Injection protocols based on CM iodine concentration of 120 and 200 mg I/mL resulted also in significantly higher CT_max_ compared to the SoC iodine concentration of 320 mg I/mL (*p*-values of 0.036 and 0.027, respectively).

**Table 2:** Mean values (N = 8) of each Figure of Merit for the vena cava with corresponding 95% confidence intervals.

CM iodine concentration (mg I/mL)	CT_max_ (HU)	∆t_>150_ (s)	TTP (s)
120	191.1*^,^** (177.7–204.5)	15.4*^,^** (10.1–20.8)	33.8*^,^** (32.8–34.7)
160	193.6** (179.2–208.0)	14.9*^,^** (11.5–18.3)	33.7*^,^** (32.2–35.2)
200	192.9*^,^** (180.8–204.9)	15.6*^,^** (11.8–19.3)	37.3** (36.2–38.4)
270	187.1** (174.2–200.0)	16.8*^,^** (9.4–24.1)	37.2*^,^** (36.0–38.4)
300	169.0 (153.2–184.8)	8.1 (0.3–15.9)	39.4 (38.2–40.6)
320	163.4 (141.4–185.4)	5.8 (0.4–11.3)	39.6 (37.5–41.7)
370	154.7 (137.9–171.5)	3.3 (−0.8–7.4)	40.7 (39.0–42.3)

*Indicates significant difference compared to SoC CM with iodine concentration of 320 mg I/mL (*p* < 0.05).

**Indicates significant difference compared to SoC CM with iodine concentration of 370 mg I/mL (*p* < 0.05).

The peak width (∆t_>150_) of the vena cava inferior was measured as the duration (s) for which CT signal remained above 75 percent from the CT_max_ or 150 HU, was significantly larger in the time enhancement curves of the iodine concentrations of 120, 160, 200 and 270 mg I/mL compared to the SoC iodine concentrations of 320 and 370 mg I/mL (*p*-values between 0.002 and 0.020) (Table 2).

As with the arterial enhancement, the TTP of the iodine concentrations of 120, 160 and 270 mg I/mL were significantly shorter compared to the TTP of the SoC iodine concentrations, 320 and 370 mg I/mL (*p*-values between 0.001 and 0.034) (Table 2). The TTP of the CM with iodine concentration of 200 mg I/mL was also significantly shorter compared to the TTP of the SoC iodine concentration of 370 mg I/mL (*p* = 0.003).

### Liver parenchyma enhancement

Also, a negative correlation between maximal enhancement in the liver parenchyma and CM iodine concentration was found (r = 0.54, *p*-value < 0.0001) at constant TID and IDR. The CT_max_ was significantly higher for the CM iodine concentrations of 120, 160 and 200 mg I/mL compared to the SoC iodine concentrations of 320 and 370 mg I/mL (*p*-values between 0.002 and 0.036) (Fig. 2C and Table 3). The CT_max_ of the CM with iodine concentration of 270 mg I/mL is also significantly higher compared to the CT_max_ of the SoC iodine concentration of 320 mg I/mL (*p* = 0.009).

**Table 3:** Mean values (N = 8) of each Figure of Merit for the liver parenchyma with corresponding 95% confidence intervals.

CM iodine concentration (mg I/mL)	CT_max_ (HU)	TTP (s)
120	109.7*^,^** (106.0–113.3)	61.8** (57.3–66.2)
160	111.0*^,^** (107.5–114.5)	62.4** (57.2–67.6)
200	110.5*^,^** (107.4–113.6)	65.9 (57.3–74.4)
270	109.4* (103.8–115.1)	61.0** (52.7–69.3)
300	105.3 (100.5–110.2)	61.9 (51.7–72.2)
320	101.4 (98.0–104.8)	67.4 (61.4–73.4)
370	103.0 (98.4–107.5)	69.1 (64.4–73.8)

*Indicates significant difference compared to SoC CM with iodine concentration of 320 mg I/mL (*p* < 0.05).

**Indicates significant difference compared to SoC CM with iodine concentration of 370 mg I/mL (*p* < 0.05).

The TTP of the CM with iodine concentrations of 120, 160 and 270 mg I/mL was significantly shorter compared to the SoC iodine concentration of 370 mg I/mL (*p*-values between 0.018 and 0.040) (Table 3).

### Viscosity measurements

Mean measured viscosities are shown in Table 4. Viscosity measurement errors (SD based on N = 3) were between 0.2% and 2.7%. Iopromide (370 mg I/mL), which has a smaller molecular weight, had a lower viscosity, comparable to that of Ioforminol at 300 mg I/mL. A weak negative correlation was found between viscosity and injection pressure (r = 0.42, *p*-value = 0.001).

**Table 4:** Injection protocol details delivering the contrast media (CM) at constant iodine delivery rate (IDR) and total iodine dose (TID) of 0.64 g I/s and 320 mg I/kg, respectively.

CM iodine concentration (mg I/mL)	Viscosity (mP.s) (±SD)	Injection pressure (PSI) (±SD)	CM injection volume (mL/kg)	CM injection rate (mL/s)
120	1.42 (±0.007)	193.6 (±28.04)	2.67	5.3
160	1.91 (±0.014)	170.1 (±29.8)	2.00	4.0
200	2.58 (±0.004)	135.3 (±20.4)	1.60	3.2
270	14.0 (±0.680)	148.5 (±18.4)	1.20	2.4
300	21.2 (±0.180)	125.0 (±10.1)	1.10	2.1
320	29.7 (±0.104)	187.0 (±40.2)	1.00	2.0
370	21.7 (±0.208)	101.5 (±20.0)	0.86	1.7

## Discussion

The objective of this preclinical animal study was to investigate the net effect of CM iodine concentration on CT enhancement at constant TID and IDR. The higher and wider peaks of the time enhancement curves observed for the low iodine concentration CM suggest that, when administered at constant TID and IDR, CM with lower iodine concentration improve enhancement. This is confirmed by the observed correlation between the maximal enhancement and CM iodine concentration, which is most pronounced in the arterial phase. For example, when comparing the arterial enhancement of 160 mg I/mL with the SoC iodine concentration of 320 mg I/mL, we observed an increase in CT_max_ of 19%, and in ∆t_>200_ of 75%. The CM with the reduced iodine concentration reached maximal enhancement faster, reflected in the TTP which was 20% shorter compared to the SoC iodine concentration. Similar but less pronounced results can be observed in the vena cava inferior and the liver parenchyma. In the vena cava inferior we observed an increase of 18% in CT_max,_ a 157% increase in ∆t_>150_ and a 15% shorter TTP in favor of 160 mg I/mL compared to the SoC iodine concentration of 320 mg I/mL. In the liver parenchyma, the CT_max_ of 160 mg I/mL is 9% higher and the TTP 7.5% shorter compared to the SoC iodine concentration of 320 mg I/mL.

As a consequence of the increased enhancement by lower CM iodine concentrations, the total iodine load could be reduced without reducing the CT signal. Our results suggest that SoC injection protocols could be replaced by an alternative injection protocol with a low iodine concentration CM at a higher injection rate, to preserve the IDR, but with a reduced TID to preserve the CT signal. There is controversy in the literature about the risk of contrast media-induced nephrotoxicity when using intravenous low-osmolality iodinated contrast material^20–22^. Iodinated contrast media are among the most commonly prescribed agents in current medical practice, with more than 30 million doses administered annually^22^ and until there is no complete understanding of the true incidence and clinical relevance of contrast-induced nephropathy (CIN), a conservative aproach on iodine dose is advised in the context of patient safety^6,7^. Recent studies also suggest an impact of iodine based contrast media on the radiation induced DNA damage^23–25^, an extra argument for iodine dose reducing measures. The larger arterial ∆t_>200_ and venous ∆t_>150_ increases the scan window flexibility, making scan timing less critical (prone to less operator errors) and ensures a more constant enhancement in long-duration CT scans covering a larger anatomical range.

In general, the data suggest that at constant TID and IDR, there is an overall trend in which image quality FoM improve significantly when the iodine concentration of the CM is reduced compared to the SoC iodine concentrations. These results are in agreement with previous studies^13–17^ who reported similar results for a limited selection of CM iodine concentrations. The observed effect is surprising in the sense that one would expect that as the TID and IDR are constant for each of the protocols, the resulting vessel and tissue enhancement would be the same. In the literature, several suggestions were made towards the origin of this effect: CM viscosity^14–16^, volume effect^17^ and dead space volume^18^. Most frequently stated is that the impact of viscosity on flow characteristics might be the explanation for the observed effect. Assuming that CM with lower iodine concentrations, typically having lower viscosity, may distribute more evenly through the blood vessels resulting in an improved arterial enhancement. However, we found no clear evidence that viscosity has a significant impact on tissue enhancement. Iopromide, with an iodine concentration of 370 mg I/mL, follows the described trend of reduced enhancement but has a lower viscosity compared to Ioforminol 320 mg I/mL. Another possible explanation, suggested by Awai and colleagues, is the dead space volume implying that a volume of the injected CM is retained between the brachial vein and the superior vena cava. This venous space can contain 20–30 mL of the injected CM which is in favour of the diluted CM with their higher injection volumes. Again, this argument does not explain the observed effect in this study, as the PACs deliver the CM directly to the right atrium, making pooling of the CM in the venous system impossible. Also injector accuracy (maximal 2% margin of error, obtained by experimental validation) can be ruled out as the reason of higher arterial enhancement of CM with lower iodine concentration. The “volume effect”, formulated by Han *et al*., is a plausible explantation because it is feasable that the administration of higher injection volumes triggers a physiologic response, like an increase in cardiac output, that has a possible impact on blood vessel and tissue enhancement. Non-invasively, cardiac output can be obtained from a cardiac CT scan^26^. Unfortunatly, in our study, it could not be derived from the CT images since our scan protocol at hepatic level did not include the heart anatomy. A similar study based on cardiac CT scans can be a topic of further research to investigate the impact of the injection protocol on cardiac output.

Although the viscosity of the diluted CM is typically lower, an impact of the increased injection rate on injection pressure can be expected, in order to maintain the constant IDR. Indeed, a negative correlation between the injection pressure and CM iodine concentration was observed (r = 0.56, *p*-value < 0.001) (injection pressure values, Table 4) but none of the injection protocols reached the institutional injection pressure limit of 300 PSI. High injection rate and -pressure are somethimes considered to increase the risk of extravasation or discomfort at the site on injection for patients with a pheripheral venous injection but no correlation between injection rate and frequency of extravasation has been discribed in literature^16,27^. Nevertheless, older patients with a reduced renal function, who might benefit the most from an iodine dose reduction, have typically a poor vascular status with an increased risk of extravasation. Therefore, we consider the possibility to further increase the injection rate and volume for the CM with even lower iodine concentrations limited.

A limitation of the study is that these observations are the result of animal experiments. The minipig model, with similar anatomy, heart function, blood circulation and effective abdominal diameter compared to humans^28–30^, offered the opportunity to consider different high dose dynamic scans within one subject, eliminating the inter-patient variability^31^. The reproducibility of the injection protocols was increased by equipping the minipigs with a PAC unit. Further validation is necessary although some groups have confirmed similar findings in patients^12–14,8^. Also, the study design was chosen to investigate the net effect of CM iodine concentrations on tissue enhancement when administered at constant TID and IDR. Other injection protocol parameters and iodine dose reducing measures, like using a lower tube voltage, were not assessed in this study. In addition, our scan protocol did not allow to obtain the cardiac output of the minipigs.

## Conclusion

Despite constant TID and IDR, injecting a reduced iodine concentration CM at higher injection rate and volume results in a higher maximal enhancement and wider time window in which the enhancement stays clinically relevant, compared to the administration of a high iodine concentration CM at lower injection rate and volume. In addition, this increase in maximal enhancement creates an opportunity to decrease the iodine dose but preserve images of diagnostic quality. An alternative clinical injection protocol could be composed with a diluted CM administered at a higher injection rate to maintain the IDR and an adapted injection volume to result in a lower TID but similar CT signal compared to a SoC injection protocol.

## Materials and Methods

### Porcine model

This study used a porcine model as minipigs have similar anatomy, heart function, blood circulation and effective abdominal diameter compared to humans^28–30^. Four healthy female, naive Göttingen minipigs (Ellegaard, Dalmose, Denmark) with a mean weight of 38.5 kg (range 36.5–40.4 kg) and a mean effective abdominal diameter of 76.7 cm (range 73–80.5 cm) were included in the study. Study approval was granted by the ethical committee for animal experiments of the Vrije Universiteit Brussel (VUB) and all experiments were performed in accordance with relevant guidelines and regulations. Two weeks before the start of imaging, a PAC unit (Power PAC II, 1.9 mm, Smiths Medical, St Paul, MN, USA) was surgically inserted subcutaneously into the left shoulder with a connection to the superior vena cava for controlled CM injections. The animals were each scanned up to twice weekly with a minimal inter-scan delay of 72 hours to avoid an iodine retention bias originating from previous scans. Anaesthesia was induced by an intramuscular injection of an anaesthetic cocktail (500 mg Zoletil-100, 6.25 mg Rompun, 1.25 mL Ketamine and 2.5 mL Dolorex) at a dose of 0.05 mL/kg.

### CM injections and imaging protocol

A broad range of CM iodine concentrations was studied based on a low-osmolar dimeric contrast agent, Ioforminol (GE-145) with 320 mg I/mL (GE Healthcare Ltd.)^32^, along with a high iodine concentration CM (Iopromide, Ultravist™, Bayer HealthCare) with 370 mg I/mL. These CM iodine concentrations were considered as the SoC iodine concentration, as they are frequently used in clinical practice. The following series of iodine concentrations was obtained by diluting Ioforminol 320 mg I/mL with saline (Baxter, Deerfield, Ill, USA): 120, 160, 200, 270 and 300 mg I/mL.

The different CM iodine concentrations were administered to the anaesthetized minipigs from a pre-defined, randomized list and imaged on a 64-slice multidetector CT scanner (Discovery 750HD, GE Healthcare, Waukesha, WI, USA). Dynamic images were acquired for 120 s at a rate of 2 images/s. X-ray tube parameters 120 kVp and 300 mA, covering a 20 mm (4 × 5 mm slices) thick transaxial slab of the liver, allowing to monitor the dynamic contrast enhancement at high frequency in the abdominal aorta, inferior vena cava and liver parenchyma.

Each of the 7 injection protocols was randomly repeated 8 times over the 4 animals resulting in a total of 56 experiments. For each injection protocol, the injection rate and injection volume were adjusted to result in a constant TID and IDR (Table 4). The TID (mg I/kg) is determined by the product of CM iodine concentration (mg I/mL) and the injection volume (mL) derived by the body weight of the minipig (kg) while the IDR (g I/s) is determined by the product of the CM iodine concentration (mg I/mL) and the injection rate (mL/s). For example when a SoC CM with an iodine concentration of 320 mg I/mL is administered at 1 mL/kg and 2 ml/s, a diluted CM with an iodine concentration of 160 mg I/mL needs to be administered at double the injection volume (2 mL/kg) and injection rate (4 mL/s) to result in the same TID of 320 mg I/kg and IDR of 0.64 g I/s. The temperature of the CM was equilibrated to the controlled room temperature of 20 °C. The CM dose was then injected into the PAC with a dual head injector (Nemoto-Kyorindo, Tokyo, Japan). At the same injection rate, each CM injection was followed by an additional 12 mL saline chaser which equals the exact volume of the dead space of the tubing from the injector to the PAC, to make sure the exact CM volume was delivered. The injection peak pressure was recorded during each injection protocol from the injector (Table 4).

### Viscosity

CM viscosity was studied as a potentially important parameter for the CM tissue distribution and kinetics. The viscosity of all the CM iodine concentrations used for imaging was measured in a laboratory environment with a set of Cannon-Fenske Routine Viscometers (Cannon Instrument Company) at 20 °C. Each measurement was repeated 3 times and mean values were calculated.

### Quantitative analysis of contrast enhancement

**Figure 6:**
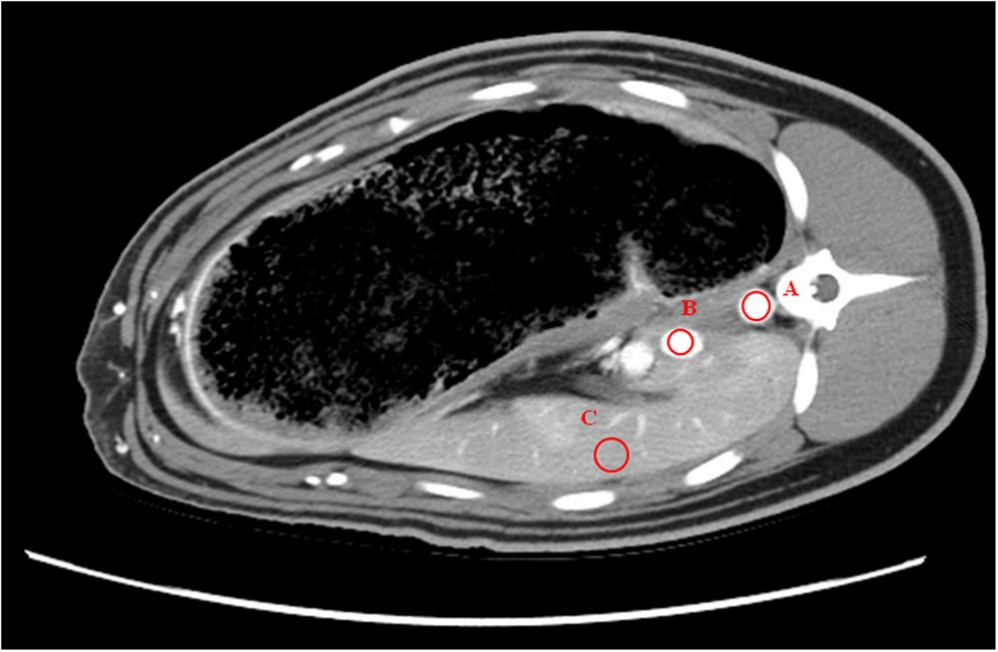
Placement of the ROI in the aorta (**A**), vena cava (**B**) and liver parenchyma (**C**).

The mean attenuation and standard deviation (SD) was measured by placing a circular, planar (2D) region of interest (ROI) in the abdominal aorta, inferior vena cava and liver parenchyma on a suitable frame of the dynamic image of 240 frames at the hepatic level (Fig. 6). ROI size was adjusted to the vascular diameter in a conservative way to avoid partial volume contributions. Liver ROIs were placed in homogenous tissue regions without visible blood vessels.

Figures of merit (FoM) were defined for each tissue of interest (Table 5). Maximal enhancement (CT_max_ (HU)) and time to peak (TTP (s)) were extracted for all tissue types. The peak width (∆t) was measured as the duration (s) for which CT signal remained above 75 percent from the CT_max_ for the aorta and vena cava inferior.

**Table 5:** Figures of Merit.

Tissue of interest	Figures of Merit		
Aorta	Maximal CT enhancement (CT_max_)	Temporal window in which the CT value remained above 200 HU (∆t_>200_)	Time to peak (TTP)
Vena cava	Maximal CT enhancement (CT_max_)	Temporal window in which the CT value remained above 150 HU (∆t_>150_)	Time to peak (TTP)
Liver parenchyma	Maximal CT enhancement (CT_max_)	Time to peak (TTP)	

### Statistical analysis

Statistical analysis was done by a nonparametric Mann-Whitney U test to compare the FoM for the different CM iodine concentrations using commercially available software (SPSS, Chicago, Ill, USA). Differences in FoM with p-values smaller than 0.05 were considered to be significant. Correlations were investigated using a Spearman Rank-Order Correlation test.

## Data Availability

The datasets generated during and analysed during the current study are available from the corresponding author on reasonable request.
